# AI/ML-based computational models for toxicity prediction

**DOI:** 10.1007/s11356-025-37354-8

**Published:** 2026-01-12

**Authors:** Sushmita Barua, Badhrinarayanan Balaji, Seetharaman Balaji

**Affiliations:** 1 Manipal Institute of Technology, Manipal Academy of Higher Education, Manipal, Karnataka 576104 India; 2 Manipal School of Information Sciences, Manipal Academy of Higher Education, Manipal, Karnataka 576104 India

**Keywords:** Ecotoxicity, Animal toxicity, Human toxicity, Computational toxicity, AI, ML, SDG 3, 6, 9, 12, 14

## Abstract

The increasing demand for accurate toxicity assessment and to minimise or eliminate the use of animal testing has accelerated the development of numerous computational models, AI/ML models, and online resources that support research in computational toxicology. This review addresses toxicity prediction and chemical safety evaluation, focusing on computational models and data coverage, Molecular Descriptors, QSAR models, AI/ML-based approaches, Explainable AI, predictive methodologies, regulatory relevance, and accessibility. This collectively enables the identification, prediction, and analysis of chemical toxicity across various biological endpoints. In addition, the review highlights AI/ML tools for predicting toxicity endpoints, such as neurotoxicity, hepatotoxicity, cardiotoxicity, genotoxicity, and environmental toxicity. Regulatory limitations vary significantly among countries and jurisdictions, exhibiting a marked absence of convergence. Current debates regarding regulatory norms focus on achieving global conformity. Regulatory adaptability is the key as AI evolves rapidly. The promotion of AI/ML tool integration and interoperable frameworks could substantially enhance the future of predictive toxicology.

## Introduction

Over the past few decades, the growing number of chemicals in the environment has become a significant challenge for regulatory frameworks aimed at safeguarding health and safety. The effect of chemicals on living organisms is a critical area of study, with far-reaching implications for human health, environmental protection, and policy-making. Around 25% of global mortality is linked to harmful chemicals (Chartres et al. 2019). Toxicity prediction is a fundamental aspect of chemical safety assessment, playing a crucial role in protecting human health and the environment. Regulatory agencies, such as the Food and Drug Administration (FDA), European Medicines Agency (EMA), and the Organisation for Economic Co-operation and Development (OECD), have established detailed guidelines to ensure the safe evaluation of pharmaceuticals, industrial chemicals, and other substances. However, traditional toxicity testing methods are often time-consuming, expensive, and ethically challenging due to the reliance on animal studies. To address these limitations, there has been a growing shift towards computational toxicity that uses artificial intelligence (AI) and machine learning (ML) to assess toxicity. Recognising this transformation, regulatory bodies are gradually adapting their frameworks to incorporate AI-based tools, emphasising transparency, explainability, and validation to ensure these technologies can reliably be integrated (Rovida et al. 2015; Amorim et al. 2024).

**Fig. 1:**
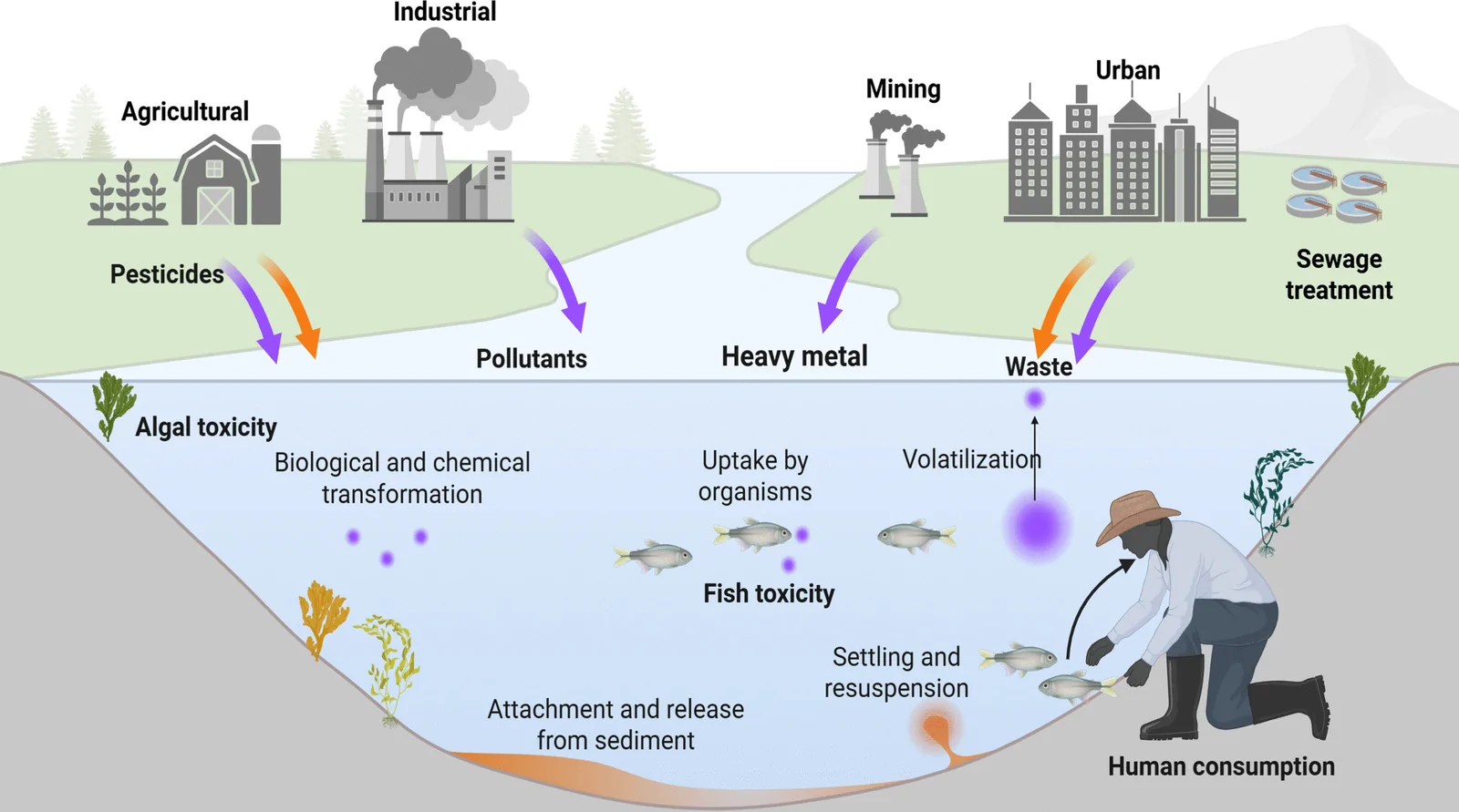
Toxicity potential of chemicals in human health and the environment

As new substances continue to emerge, evaluating the toxicity of chemicals is crucial for assessing their risks to human health and environmental safety (Persad and Bobst 2020). Toxicity may be present in multiple forms and can be evaluated either quantitatively using metrics such as the lethal dose 50 (LD₅₀) or qualitatively by categorising it into levels such as low, moderate, or high toxicity. The ‘No Observed Effect Level’ (NOEL) represents the highest dose at which no noticeable harmful effects are observed. NOEL serves as a key parameter in toxicology and risk assessment for determining safe exposure levels (Fuadah et al. 2024; Baird et al. 2019). Food additives are commonly used to enhance the quality and shelf life of food products, but they may pose health risks through the release of harmful chemicals (Fig. 1).

For instance, azodicarbonamide (ADA) is a dough-enhancing agent approved in Nigeria as a substitute for potassium bromate, which is banned in several countries due to its nephrotoxic effects. ADA is also used in countries like the United States as a leavening agent in baked foods. However, it has been identified as a respiratory sensitiser in industrial settings, prompting its ban in the European Union. Limited animal studies have reported mild kidney and haematological toxicity following acute oral exposure to ADA (Olofinnade et al. 2020). A major concern is that regulations differ widely across countries and jurisdictions, with a notable lack of convergence.

Besides ADA, there are more concerning substances, including bisphenol A (BPA), which is commonly found in plastics and food container linings and is known to be an endocrine disruptor linked to fertility issues, developmental abnormalities, and hormone-related cancers (Hafezi and Abdel-Rahman 2019). In the construction industry, additives such as phthalates (including dibutyl phthalate and diethyl phthalate) are commonly used to enhance flexibility and performance. These additives, along with plastic fragments of various sizes and shapes, are found across all environmental compartments. Phthalates can enter the human body through ingestion, inhalation, or skin contact and have been detected in blood, amniotic fluid, and urine. They play a significant role as endocrine disruptors, affecting erythrocytes, and influence reproductive health (Arrigo et al. 2023).

Pesticides such as malathion, chlorpyrifos, and bromoxynil are chemicals used to protect crops and control pests. While they help increase food production and manage plant diseases, they also pose serious risks to both people and the environment (Table 1). Over time, these chemicals find their way into the air we inhale, the water we consume, the land utilised for agriculture, and even the food itself, resulting in acute toxicity and chronic diseases (Ahmad et al. 2024).

**Table 1:** Chemicals with exposure dosage  demonstrating specific toxicological endpoints

Sl. No.	Chemical	Species	Route	Dose	Endpoint	References
1	Dioxin	Rodent	Oral	025–1.0 μg/kg	Neurotoxicity, Reproductive Toxicity	(Zhu et al. 2009)
2	Bisphenol A	Rodent	Oral	50–500 μg/kg	Neurotoxicity	(Kuwahara et al. 2013)
3	Malathion	Rat	Oral	2100 mg/kg	Immunotoxicity	(Pupim et al. 2023)
4	Dichlorvos	Rat	Oral	2.5 mg/kg	Neurotoxicity	(Okoroiwu and Iwara 2018)
5	Cyproconazole	Rat	Oral	1620 mg/kg	Carcinogenic	(Karmaus et al. 2022)
6	Phthalates	rodent	Oral	1–30 g/kg	Hepatotoxicity, Nephrotoxicity	(Heudorf et al. 2007)
7	Anthracene	Mice	Interparental	> 430 mg/kg	Respiratory Toxicity	(Valizadeh-Kakhki et al. 2014)
8	Atrazine	Rat	Oral	672 mg/kg	Myotoxicity Respiratory Toxicity	(Ghosh and Philip 2013)
9	Monosodium glutamate	Rat	Oral	16,600 mg/kg	Neurotoxicity	(AAT Bioquest Inc. 2025)
10	Stevioside	mice	Oral	15,000 mg/kg	Nephrotoxicity	(Wang et al. 2025)
11	Glyphosate	Rat	Oral	10,537 mg/kg	Carcinogenic	(Martínez et al. 2018)
12	Cyanuric acid	Rat	Oral	7700 mg/kg	Reproductive Toxicity	(Sun et al. 2016)
13	Melamine	Rat	Oral	6000 mg/kg	Reproductive Toxicity	(Sun et al. 2016)
14	Delta-9-tetrahydrocannabinol (THC)	Rat	Oral	1270 mg/kg	Hepatotoxicity	(Černe 2020)
15	Trinitrotoluene (TNT)	Rat	Oral	794 mg/kg	Cellular Toxicity	(Gromek et al. 2022)
16	Solanine	Human	Oral	2.8 mg/kg	Enterotoxicity, Neurotoxicity	(Vasquez et al. 2019)
17	Sodium nitrite (NaNO_2_)	Rat	Oral	180 mg/kg	Cytotoxicity	(Ansari et al. 2017)
18	Methylenedioxymethamphetamine (MDMA)	Rat	Oral	160 mg/kg	Neurotoxic	(Mueller et al. 2013)
19	Uranyl acetate dihydrate (UO_2_(CH_3_COO)_2_)	Mouse	Oral	136 mg/kg	Nephrotoxicity	(Majeed et al. 2010)
20	Dichlorodiphenyltrichloroethane (DDT)	Mouse	Oral	135 mg/kg	Neurotoxicity	(Kaushik and Pundir 2025)
21	Thiopental sodium	Rat	Oral	64 mg/kg	Systemic Toxicity	(Karmaus et al. 2022)
22	Chloroxynil	Rat	Oral	200 mg/kg	Mammalian toxicity	(Lovecka et al. 2015)
23	Carfentanil	Rat	Intravenous	3.39 mg/kg	Respiratory Toxicity, Cardiotoxicity	(Zawilska et al. 2021)
24	Strychnine	Human	Oral	1–2 mg/kg	Neurotoxic	(Patocka 2020)
25	Bufotoxin	Cat	Intravenous	300 μg/kg	Cardiotoxicity	(Dalefield 2017)
26	Methamphetamine	Rat	Intraperitoneal	57 mg/kg	Hepatotoxicity, Nephrotoxicity, Hematotoxicity	(Kiyatkin and Sharma 2009)
27	Aconitine	Human	Oral	1–2 mg/kg	Neurotoxicity Cardiotoxicity	(Gao et al. 2020)
28	Cantharidin	Human	Oral	500 μg/kg	Hepatotoxicity	(Jin et al. 2023)
29	Sarin	Mouse	Subcutaneous	172 μg/kg	Neurotoxicity	(Hanak et al. 2025)
30	Aflatoxin B1	Rat	Oral	480 μg/kg	Hepatotoxicity, Cardiotoxicity	(Altyar et al. 2023)
31	Bromoxynil	Fish	Exposure	0.4 mg/L	Hepatotoxicity	(Afzal et al. 2024)
32	Ioxynil	Rat	Oral	110–230 mg/kg	Mammalian toxicity	(Lovecka et al. 2015)
33	Tolfenpyrad	Rat	Oral	150 mg/kg	Acute oral toxicity	(Promrungsri et al. 2024)
34	Triazophos	Rat	Oral	82 mg/kg	Neurotoxicity, Reproductive Toxicity	(Sharma and Sangha 2014)
35	Brodifacoum	Rat	Oral	270 μg/kg	Neurotoxicity	(Kalinin et al. 2017)
36	Ricin	Mice	Intravenous	2–8 µg/kg	Hepatotoxicity	(Worbs et al. 2011)
37	Cyfluthrin	Rats	Oral	20 mg/kg	Neurotoxicity	(Rodríguez et al. 2016)
38	Dimethoate	Mice	Oral	150 mg/kg	Cellular Toxicity, Neurotoxicity, Cardiac Toxicity, Pulmonary Toxicity, Hepatoxicity, Pancreatic Toxicity, Reproductive Toxicity, Environmental Toxicity	(Eken 2021)
39	Endosulfan	Rats	Oral	80 mg/kg	Neurotoxicity	(Raina et al. 2013)
40	Cypermethrin	Rats	Oral	250 mg/kg	Neurotoxicity	(Raina et al. 2013)
41	Methomyl	Bird	Oral	24.2 mg/kg	Respiratory toxicity	(Mortensen and Serex 2014)
42	Fipronil	Rat	Oral	91 mg/kg	Environmental Toxicity, Hepatoxicity, Neurotoxicity, Nephrotoxicity, Reproductive Toxicity, Cellular Toxicity	(Bhatt et al. 2023)
43	Pyrethroid	Human	Inhalation	250 mg/kg	Nephron Toxicity, Pulmonary Toxicity,	(Ramchandra and Victor 2019)
44	Diazinon	Zebrafish	Oral	16 mg/kg	Fish Toxicity, Immunotoxicity, And Reproductive Toxicity	(Darvishi et al. 2022)
45	Imidacloprid	Rat	oral	20 mg/kg	Immunotoxicity, Neurotoxicity, Reproductive Toxicity, And Environmental Toxicity	(Kapoor et al. 2010)
46	Clothianidin	Rat	Oral	20 mg/kg	Nephrotoxicity, Genotoxicity, And Environmental Toxicity	(Ozsahin et al. 2014)
45	Imidacloprid	Rat	Oral	475 mg/kg	Neurotoxicity, Environmental Toxicity	(Mohamed et al. 2009)
46	Phosphamidon	Rat	Intraperitoneal	2 mg/kg	Hepatoxicity	(Alsherbiny et al. 2019)
47	Resmethrin	Fish	Oral	0.22–15.0 μg/L	Fish Toxicity, Environmental Toxicity	(Jackson et al. 2008)
48	Tau-fluvalinate	Gammarus roeseli	Exposure	17.29 μg/L	Genotoxicity	(Sari 2022)
50	Acephate	Mice	Intraperitoneal	361 mg/kg	Neurotoxicity, Developmental Toxicity	(Takayasu et al. 2019)
51	Omethoate	Rat	Oral	150 mg/kg	Cellular Toxicity, Cardiac Toxicity	(Mirajkar 2014)
52	Phorate	Mice	Oral	8.0 mg/kg	Bird Toxicity, Fish Toxicity, And Environmental Toxicity,	(Simonelli et al. 2022)
53	Oxamyl	Rat	Oral	2.5 to 3.1 mg/kg	Developmental Toxicity, Ophthalmal Toxicity, Dermal Toxicity, Aquatic Toxicity, Environmental Toxicity	(Biancardi et al. 2022)
54	Nitrofen	Rat	Oral	121 mg/kg	Environmental Toxicity, Aquatic Toxicty	(Iyer and Makris 2010)
55	Aldicarb	Rat	Oral	1 mg/kg	Neurotoxicity, Developmental Toxicity, And Reproductive Toxicity	(Blacker et al. 2010)
56	Permethrin	Rat	Oral	150 mg/kg	Neurotoxicity, Environmental Toxicity	(López-Aceves et al. 2021)
57	Aldrin	Rat	Oral	4000 mg/kg	Environmental Toxicity, Aquatic Toxicity, Neurotoxicity	(Chen 2014)
58	Heptachlor	Rat	Oral	70 to 230 mg/kg	Environmental Toxicity, Aquatic Toxicity, Reproductive Toxicity, Developmental Toxicity	(Reed and Koshlukova 2014)
59	Fenarimol	Mice	Oral	200 μg/kg	Endocrine Disrupter	(Park et al. 2011)
60	Mancozeb	Rat	Oral	> 5,000 mg/kg	Neurotoxicity	(Abdourahime et al. 2020)

In 2022, global agricultural pesticide consumption totalled 3.70 million tons. Asia emerged as the largest exporter, supplying 3.5 million tons valued at approximately USD 21.7 billion. Globally, an estimated 385 million cases of unintentional acute pesticide poisoning occur each year, leading to approximately 11,000 deaths. Considering the global agricultural workforce of 860 million, nearly 44% of farmers are affected by pesticide poisoning annually. In developing nations alone, contaminated food is responsible for the deaths of almost 20,000 people each year (Garud et al. 2024).

**Fig. 2:**
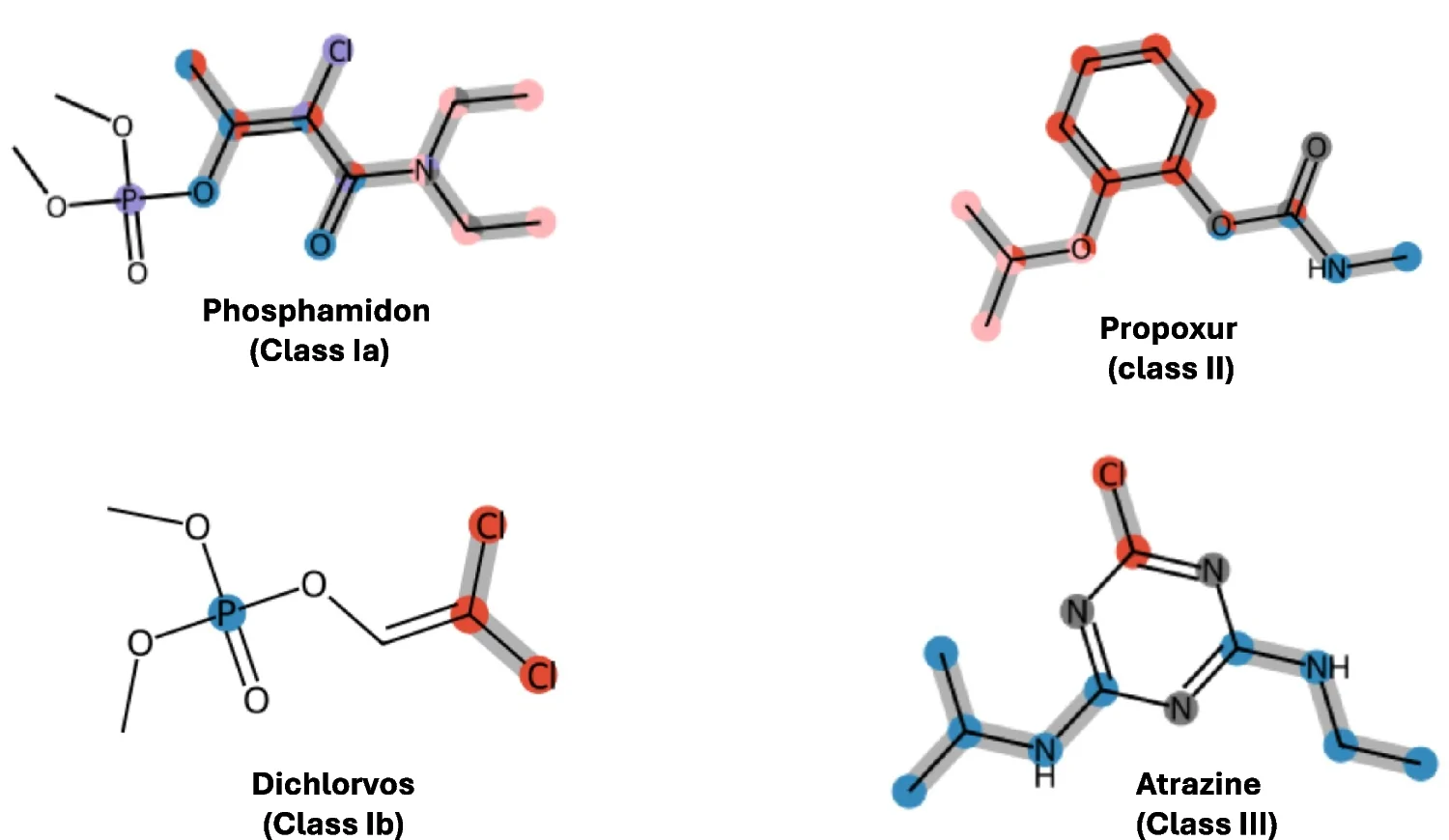
The World Health Organisation (WHO) classifies chemicals based on their toxicity, as extremely hazardous (class I-a), highly hazardous (class I-b), moderately hazardous (class II), and slightly hazardous (class III). Examples for each class are shown

According to the World Health Organisation (WHO), approximately one million individuals are affected annually by acute pesticide poisoning. Reported mortality rates range between 0.4% and 1.9% each year, with occupational exposure accounting for nearly 70% of these deaths. In addition, prolonged exposure to low doses of pesticides has been linked to a range of medium- and long-term health outcomes, including various cancers and neurological disorders (Hassaan and El Nemr 2020). The WHO classification of chemicals based on toxicity is shown in Fig. 2.

Artificial Intelligence and Machine Learning (AI/ML) models have significantly gained attention for their ability to analyse large datasets and identify complex patterns related to chemical toxicity. These models can be used to detect toxicity for the protection of one’s health and the environment (Lin and Chou 2022). These models offer accurate, cost-effective alternatives to traditional testing methods, significantly contributing to the minimisation or elimination of animal testing in scientific procedures. The AI/ML models can predict multiple forms of toxicity, ranging from cellular to organ-level (Ajisafe et al. 2025). By simulating toxicological responses, these approaches accelerate the risk assessment while endorsing ethical and regulatory efforts to reduce animal use in research (Dent et al. 2018). The implementation of computational models facilitates Next-Generation Risk Assessment (NGRA), which advocates for ethical and human-relevant approaches to chemical safety evaluation (Baltazar et al. 2020; Han et al. 2024). This review is an attempt to address toxicity prediction and chemical safety evaluation with a focus on computational models, Molecular Descriptors, QSAR models, AI/ML-based approaches, and Explainable AI. Ideally, integrating predictive toxicological approaches with regulatory requirements and ethical AI considerations is an essential aspect to support strong scientific standards.

## Databases and datasets

Databases are crucial for toxicity prediction as they provide the chemical, biological, and toxicological data necessary to train and validate computational models. These databases comprise comprehensive information such as chemical structures, physicochemical properties, and experimentally measured toxicity endpoints. They enable the development of reliable QSAR models and machine learning algorithms by offering curated, standardised, and biologically relevant datasets. For instance, Tox21, ToxCast, PubChem, DSSTox, and REACH contain high-throughput screening data and regulatory submissions that support both academic research and industrial applications. By facilitating large-scale data access, these resources enhance model accuracy, generalizability, and reproducibility, making them indispensable tools in modern toxicology. Toxicological databases enable the rapid screening of toxic chemicals, significantly accelerating drug discovery. These databases can enhance the efficiency of conventional methods (Hevener 2018) and also improve toxicity prediction by leveraging computational techniques, statistical and AI/ML models (Singh et al. 2016).

From an ethical perspective, the growing use of datasets brings several significant concerns, including transparency, data privacy, algorithmic bias, and accessibility. Maintaining high-quality, unbiased data is also crucial, as noisy or biased inputs may lead to inaccurate predictions and potentially unsafe decisions. One of the fundamental challenges is data reproducibility, which should be available in a structured and reusable format (Murikah et al. 2024). A noticeable step towards combating this challenge is the introduction of Findable, Accessible, Interoperable, Reusable (FAIR) principles. FAIR has significantly influenced research data management since its introduction. This structured framework, FAIR, improves data management, making it easier for humans and machines to locate, access, and use data effectively (García-Closas et al. 2023).

The U.S. Environmental Protection Agency (EPA) website (https://www.epa.gov/) lists several ‘Computational Toxicology and Exposure Online Resources’ under CompTox-Tools.

a ECOTOX Knowledgebase
  The Ecotoxicology (ECOTOX) Knowledgebase is a comprehensive resource providing data on the harmful impacts of specific chemical stressors on ecologically significant aquatic and terrestrial organisms.
b ChemExpo Knowledgebase
  The Chemical Exposure Knowledgebase (ChemExpo) serves as an interactive platform for exploring and retrieving data on the application of chemicals within commercial and consumer products. Its cheminformatics modules provide both empirical and computationally predicted physicochemical properties, insights into environmental fate and transport, as well as associated toxicological information.
c CompTox Chemicals Dashboard
  The CompTox Chemicals Dashboard provides publicly available data on the chemistry, toxicity, and exposure of over one million chemicals. This information encompasses physicochemical data, hazard data, and additional details.
d GenRA tool
  The Generalized ReadAcross (GenRA) tool employs an algorithmic approach to enable systematic and reproducible predictions of in vivo toxicity and in vitro bioactivity using read-across methodologies.
e The SeqAPASS tool
  The Sequence Alignment to Predict Across Species Susceptibility tool is a rapid online screening platform that allows researchers and regulators to extrapolate toxicity data across multiple species.

Along with the EPA-listed resources, other online resources are listed in Table 2.

**Table 2:** Toxicity databases for human health and environment

S. No.	Databases	Description	Number of compounds available	URL	Reference
1	CTD (Comparative Toxicogenomics Database)	It integrates curated cross-species data to link chemicals, genes, and diseases, now expanded with CTD Tetramers and enhanced exposure-anatomy mapping to support environmental health research and hypothesis generation	17,700	https://ngdc.cncb.ac.cn/databasecommons/database/id/754	(Davis et al. 2023)
2	ECOTOX	It is the world’s largest curated ecotoxicity database, offering over one million test results with systematic review-based curation, and enhanced data accessibility, interoperability, and visualisation tools for environmental assessment and research	1 M	http://www.epa.gov/ecotox	(Olker et al. 2022)
3	DSSTox	Enhancing chemical structure-data accuracy for environmental research and supporting EPA programs through trusted public data integration	750,000	https://comptox.epa.gov/dashboard	(Grulke et al. 2019)
4	TOXRIC	A comprehensive database contains toxicological data for an ML-based prediction model	113,372	http://toxric.bioinforai.tech/	(Wu et al. 2023)
5	ToxValDB	It is a U.S. EPA-developed database designed to curate and standardise human health-relevant toxicity data. With data from 36 sources, the database supports regulatory assessments, chemical prioritisation, and modeling for human health risk evaluation and New Approach Methods (NAMs)	41,769	https://github.com/usepa/toxvaldbmain/	(Wall et al. 2025)
6	HepatoToxicity Portal (HTP)	An advanced platform that integrates curated data from multiple sources with deep learning models to predict hepatotoxicity risks	183,523	https://kobic.re.kr/htp/	(Han et al. 2025)
7	Liver tox	A free online resource offering comprehensive, up-to-date clinical information on liver injury caused by medications, herbal, and supplements	26,000	http://LiverTox.nih.gov	(Serrano 2014)

## Computational models in toxicity prediction

Computational models in toxicology provide powerful alternatives to traditional laboratory-based toxicity testing by predicting harmful effects of chemicals using mathematical algorithms and digital data. Among these, QSTR models are widely used to relate a compound’s structural and physicochemical properties to toxicity. Read-across and category-based methods extend predictions to untested chemicals based on similarities with known substances. Pharmacokinetic models, such as Absorption, Distribution, Metabolism, Excretion, and Toxicity (ADMET) models, simulate how chemicals behave in the body, offering valuable insights into exposure and dose-response. These models allow for rapid screening and prioritisation of potentially toxic compounds, significantly reducing the need for animal testing and accelerating safety evaluations in both pharmaceutical and environmental contexts.

- Ecotoxicity models
  Ecotoxicity prediction tools are essential in environmental protection, as they provide valuable data for assessing the risks associated with chemical exposure. This information is crucial for monitoring environmental pollution and developing effective strategies aimed at environmental conservation and restoration. Moreover, ecotoxicity prediction tools enhance our understanding of chemical behaviour and its ecological impacts. Human-induced chemicals in the environment serve as key drivers of environmental hazards, significantly affecting human well-being and exerting adverse impacts on the ecological conditions of affected regions (Gunjyal et al. 2023).
  Ecotoxicity: Ecotoxicity (HC50) refers to the potential of a chemical substance or physical agent to exert harmful effects on the environment and living organisms, including fish, insects, microorganisms, wildlife, and plants (Roy & Kar, 2016). An autoencoder model has been developed by Gao et al., 2022) for predicting HC50 values. This model performs nonlinear dimensionality reduction and extracts meaningful latent chemical representations from the input data, offering a distinct approach to handling redundant, high-dimensional information compared to techniques like PCA, MDS, and UMAP. These derived representations enable substantial dimensionality reduction, thereby enhancing the accuracy of chemical ecotoxicity predictions over methods that rely directly on raw input features. For more information on ecotoxicity, refer to the EPA Series 850, Ecological Effects Test Guidelines.
  Aquatic Toxicity: The increasing global contamination of freshwater systems with numerous industrial chemical compounds poses a significant aquatic environmental challenge. Although many of these substances are present at low concentrations, their occurrence, particularly as components of complex mixtures, raises serious concerns regarding both toxicological risks and aquatic toxicity (Schwarzenbach et al., 2006). According to REACH registration, the substances are classified under the aquatic environmental hazard category as harmful to aquatic life with long-term effects from chronic exposure (CLP-GHS H412) (Andreassen et al., 2025). A web-based platform, AquaticTox, predicts the toxicities and modes of action of compounds in five aquatic species, such as Oncorhynchus mykiss, Pimephales promelas, Daphnia magna, Pseudokirchneriella subcapitata, and Tetrahymena pyriformis, using ensemble models. It enables users to input chemical structures for rapid predictions and provides free access to curated toxicity data (Shi et al., 2024).
   Algal toxicity: Algal toxicity refers to the harmful impact of chemicals or pollutants on algae, which serve as primary producers in aquatic ecosystems. Algal toxicity refers to the detrimental effects of chemicals or pollutants on algae. Pesticides can have adverse effects on algae, and prolonged exposure may trigger tolerance mechanisms (Rydh Stenström et al., 2021). To predict the toxicity of chemical derivatives on algal species, robust Quantitative Structure–Activity Relationship (QSAR) models were developed using partial least squares (PLS) regression. Incorporating 2-D descriptors such as Extended Topochemical Atom (ETA) and non-ETA, the models demonstrated strong validation metrics. It also indicates that structural features such as ortho-positioned nitro groups, para-chlorine substituents, oxygen and nitrogen atoms at specific topological distances, aliphatic side chains, and others, contribute to toxicity (Seth & Roy, 2020). For more information on algal toxicity, refer to the regulatory guidelines, OECD Test Guideline No. 201 AQ(OECD 2011).
- Animal toxicity models
  Experimental approaches are often costly and time-consuming, which can delay the research process and the timely availability of results. Toxicity prediction tools allow rapid access to essential data on the adverse effects of chemicals which are exposed to both the environment and human health. Although numerous experimental methods are currently available for toxicity assessment, they are not universally applicable across all species and may fail to accurately capture environmental and health-related risks (Cunningham et al. 2010). Due to these limitations, we are moving towards computational and AI-based approaches that are increasingly enhancing the efficiency and accuracy of toxicity prediction.
   Fish toxicity: Like humans and other animal species, fish have been reported to accumulate a wide range of chemicals, including pesticides such as Propoxur, Deltamethrin, Atrazine, and Furadan, as well as food additives, and various other pollutants or contaminants. Exposure to these chemicals can result in toxicity leading to the mortality of aquatic organisms such as fathead minnow (Pimephales promelas), water flea (Daphnia magna), and Tetrahymena (Tetrahymena pyriformis) (Dong and Moon 2025; Ogeleka et al. 2016). A machine learning model, MS2Tox, predicts fish toxicity (LC50) from high-resolution tandem mass spectra without requiring full chemical identification. Trained on a dataset of 647 LC50 values from the CompTox database and validated on 219 chemicals with 420 spectra from the Mass Bank, the model achieved a low prediction error and accurately estimated LC50 values for multiple chemicals in water samples. MS2Tox outperformed simpler mass-based models, providing a fast method to identify potentially toxic compounds in water samples (Peets et al., 2022). According to OECD TG 203, the acute toxicity of a substance can be evaluated by observing its lethal effects on juvenile fish over a 96-h exposure period, as updated in the 2019 version of the guideline.
  Rat toxicity: The toxicity of chemicals varies based on the structure, mechanism of action, dosage and mortality. For instance, chemical agents like yellow phosphorus have high mortality for rodents (Jayakaran and Abhilash 2019). A web-based computational framework, PredAOT, is designed to predict the acute oral toxicity (AOT) of compounds in mice and rats. The tool initially classifies compounds as ‘toxic’ or ‘less/non-toxic’ and subsequently estimates their LD₅₀ values using a regression model. This tool was developed using acute oral toxicity (AOT) data from 6,226 compounds in mice and 6238 compounds in rats. A key strength of PredAOT lies in its ability to predict AOT for both species concurrently, with predictive performance comparable to or exceeding that of current state-of-the-art tools (Ryu et al. 2023). According to OECD TG 452 specifies that chronic toxicity testing can be performed via oral, dermal, or inhalation routes.
- Organ toxicity models
  The potential toxicological effects on organs such as neurotoxicity, pulmonotoxicity, hepatotoxicity, dermal toxicity, enterotoxicity, immunotoxicity, cytotoxicity, genotoxicity, ocular toxicity, endocrine toxicity, cardiotoxicity, renal toxicity, reproductive toxicity, myotoxicity, and osteotoxicity (Fig. 3). These can be predicted using computational toxicity prediction models that emphasise on evaluating toxicity in multiple biological systems for comprehensive risk assessment.
  **Fig. 3:**
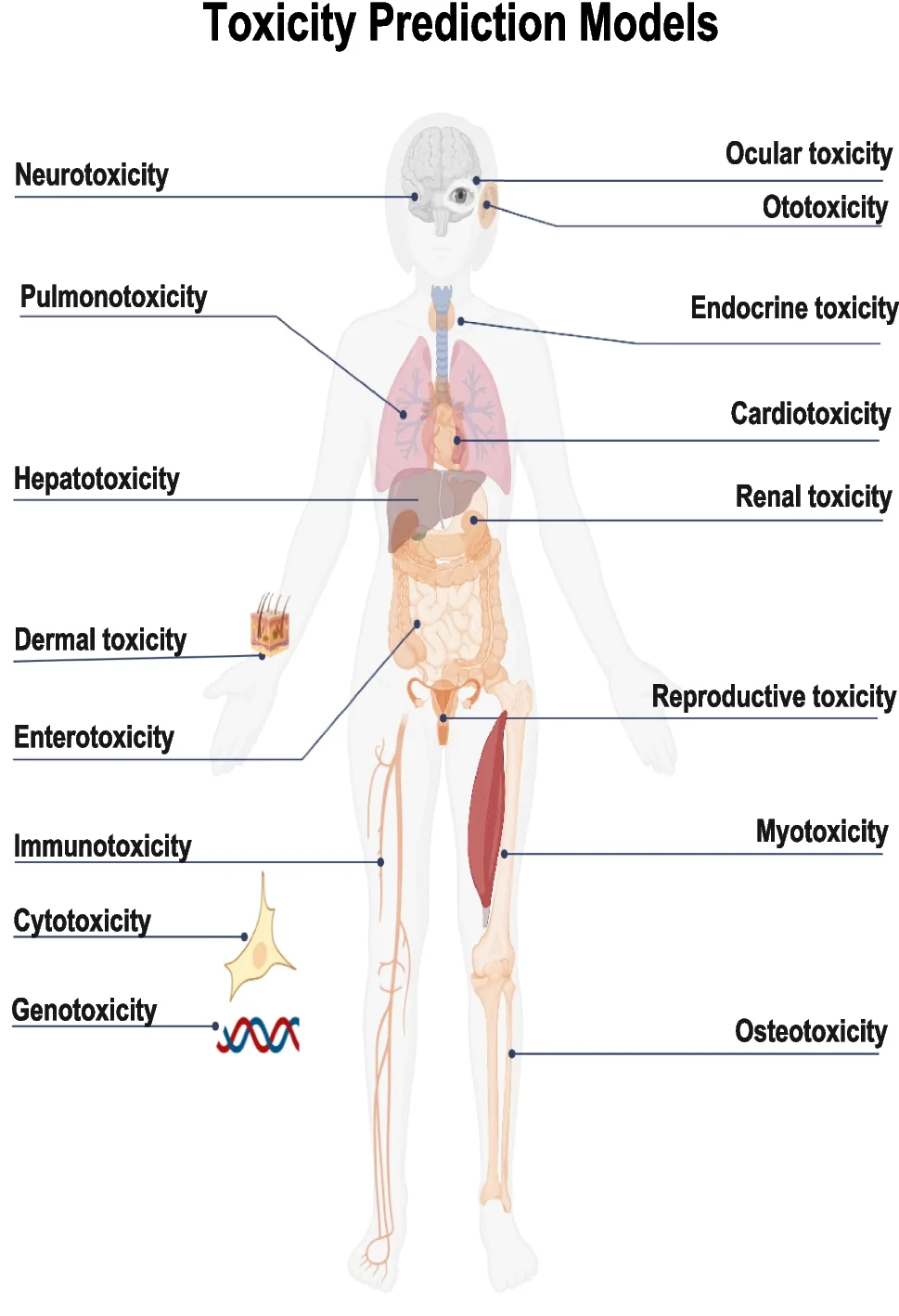
Toxicity prediction models associated with human organ systems

### Neurotoxicity

Chemicals capable of disrupting the mammalian nervous system may be synthetically produced and referred to as neurotoxicants. Neurotoxicity typically refers only when concentrations in neural tissue exceed a specific threshold. In addition to biological sources, numerous environmental pollutants, such as industrial chemicals, e.g. toluene, are proven contributors to neurodevelopmental disorders like autism, ADHD, intellectual disability, and cerebral palsy (Grandjean and Landrigan 2006; Soleimani et al. 2016; Spencer and Lein 2014). NeuTox 2.0 is a multimodal feature fusion model that integrates data on blood–brain barrier permeability, neuronal cytotoxicity, neural activity, and mammalian neurotoxicity to predict neurotoxic compounds. By combining molecular features with fine-tuned, self-supervised learning, it outperforms baseline models, particularly on small datasets. This transfer learning-based framework enhances high-throughput virtual screening and offers a more comprehensive approach to neurotoxicity prediction, addressing data limitations in environmental toxicity assessment (Pang et al. 2025). For more information on neurotoxicity, refer to the EPA, which first introduced its developmental neurotoxicity (DNT) testing guideline (83-6) in 1991 to assess the potential neurotoxic effects of certain chemicals and pesticides. Following the establishment of the Food Quality Protection Act in 1996, which aimed to enhance the protection of children’s health, the demand for DNT studies increased significantly, particularly for pesticide evaluation. In response, the EPA updated and revised its DNT guideline in 1998 (OPTS 870.6300). Later, in 2007, OECD adopted its own DNT testing guideline (TG 426), incorporating further methodological improvements (Tsuji and Crofton 2012).

### Dermal toxicity

The skin plays a vital protective role against harmful toxic chemicals, including reactive oxidants and free radicals (carbon monoxide, reactive nitrogen species, and electrophilic aldehydes), which cause oxidative stress, weaken antioxidant defences, and trigger skin tissue damage. These chemicals also increase skin water loss, degrade connective tissue, and activate enzymes that break down collagen and elastic fibres (Kathuria et al. 2017; Li Pomi et al. 2025). A novel web-based prediction model, Pred-Skin (3.0 version), for rapidly and accurately predicting skin sensitisation potential using rigorously validated QSAR models built on human, murine, and non-animal data. It provides binary predictions for skin sensitisation, Bayesian consensus results for human effects, and probability maps showing the contribution of chemical fragments. Notably, it is the first tool to incorporate human data and multiclass predictions for LLNA assays, achieving up to 89% accuracy in predicting human responses (Borba et al. 2021). The user-friendly platform requires no programming skills and is freely accessible at  https://github.com/rcbraga/pred-skin. According to the OECD Guidelines, dermal toxicity assessment is primarily conducted through Test No. 402 for acute dermal toxicity and Test No. 404 for acute dermal irritation.

### Ocular toxicity

The ocular surface, comprising the cornea, limbus, conjunctiva, and tear film, is highly vulnerable to environmental hazards such as pesticides, chemicals, and pollutants. Exposure can cause irritation and damage, including edema, inflammation, and epithelial degradation, while severe or prolonged exposure may lead to chronic conditions like dry eye disease, corneal ulcers, cataracts, glaucoma, or permanent vision loss (Rauchman et al. 2023)**.** StopTox is a freely accessible web portal that offers computational models as an alternative to in vivo 6-pack tests for predicting chemical toxicity hazards. It has been developed using OECD-compliant QSAR modelling practices and the largest publicly curated datasets for all 6‑pack assays, enabling accurate and transparent predictions across all endpoints, including eye and skin. While limited in predicting potency classes, the models are reliable for early-stage applications, such as drug discovery. STopTox (https://stoptox.mml.unc.edu/) stands out for its extensive data curation, validated statistical accuracy, and public availability, making it a valuable tool for regulators and industries to screen chemicals inexpensively and efficiently (Borba et al. 2022). For more information on ocular toxicity, refer to OECD, Test No. 405: Acute Eye Irritation/Corrosion.

### Renal toxicity

Renal toxicity is rapid decline in kidney function caused by the toxic effects of drugs or chemicals. It can occur through various mechanisms, including tubular toxicity, inflammation, glomerular damage, crystal nephropathy, and thrombotic microangiopathy (Al-Kuraishy et al. 2019). CORAL software (http://www.insilico.eu/coral) offers an alternative approach to computational modelling for toxicity prediction. Unlike traditional methods that require calculating molecular descriptors, CORAL directly utilises the molecular formula of potential toxicants in the form of SMILES (Toropov et al. 2023). For more information on renal toxicity, refer to KDIGO 2024 Clinical Practice Guideline (Stevens et al. 2024).

### Hepatotoxicity

This toxicity, caused by drugs, chemicals, or environmental agents, involves mechanisms such as inflammation, immunomodulation, and oxidative stress. Environmental chemicals, such as polychlorinated biphenyls , dioxins, and mercury, pose widespread health hazards. These exposures are linked to adverse health effects, including liver damage (Christensen et al. 2013). Bioartificial liver technology, combined with mechanistic mathematical models simulating metabolism, fluid flow, and nutrient distribution, offers a promising approach to better mimic in vivo conditions. These models enable hypothesis testing, guide experiment design, and can reduce animal testing, improving drug safety assessment and advancing predictive toxicology (Williams et al. 2013). Similarly, ToxSTAR is the first web-based platform for predicting drug-induced liver disorders such as cholestasis, cirrhosis, hepatitis, and steatosis. Its models are specifically designed to evaluate drug metabolite structures, enabling the identification of metabolites associated with liver diseases. Additionally, it incorporates applicability domain analysis to evaluate the reliability of its predictions and provides Drug-Induced Liver Injury (DILI) labels and scores for the five compounds most structurally similar to the input molecule (Shin et al. 2022). Moreover, integrating Bayesian machine learning models will improve hepatic safety risk prediction. These models achieved a balanced accuracy of 86%, with a sensitivity of 87%, a specificity of 85%, a positive predictive value of 92%, and a negative predictive value of 78%, making it useful for enhancing DILI risk assessment (Williams et al. 2020). For more information on hepatotoxicity, refer to EASL Clinical Practice Guidelines (Andrade et al. 2019) and AASLD practice guidance (Fontana et al. 2023).

### Pulmonary toxicity

The respiratory system is both a target for toxicity and a key route for absorption of inhaled gases and particles. Lung toxicity varies with dose and exposure type, causing tissue damage, functional deficits, or lesions. Upper airways and gas exchange regions respond differently to toxicants, with oxidants, harmful gases, and particles leading to inflammation, fibrosis, emphysema, or cancer. Predicting chemical-induced respiratory toxicity using molecular descriptors and term frequency–inverse document frequency (TF-IDF) features combined with machine learning algorithms. Class imbalance was addressed using the synthetic minority over-sampling technique (SMOTE), and hyperparameters were optimised with OPTUNA, a predictive model. The random forest model achieved 88.6% accuracy (AUC 93.2%) in internal validation and 92.2% accuracy (AUC 97%) in external validation, outperforming previous approaches (Shehab et al. 2025). According to OECD, test guideline No. 403 demonstrate acute inhalation toxicity.

### Cardiac toxicity

Cardiac toxicity refers to injury or functional impairment of the heart muscle (myocardium) caused by exposure to harmful agents, including certain drugs, chemicals, or environmental substances. It is estimated that at least 23% of cardiovascular disorders are linked to environmental exposures, particularly chemicals. However, the specific causative agents remain largely unidentified (Sirenko et al. 2017). A deep learning-based framework, CardioToxNet, for predicting cardiotoxicity by classifying drug-like molecules as hERG blockers or non-blockers. Using a two-step process combining multiple feature types and meta-modeling, it achieved robust performance across three independent test sets, outperforming existing methods (Karim et al. 2021). Similarly, Pred-hERG is a fast, user-friendly web server for predicting potential hERG blockers and non-blockers, requiring no programming expertise (Braga et al. 2015). For more information on cardiac toxicity, refer to the Spanish Society of Medical Oncology (SEOM) clinical guidelines (Virizuela et al. 2018).

### Ototoxicity

This is due to exposure to certain drugs or chemicals that damage the inner ear structures or auditory nerve. This condition may lead to hearing impairment, ringing in the ears (tinnitus), or issues with balance (Ganesan et al. 2018). A simple prediction model was developed to assess the risk of ototoxicity. The model uses logistic regression, readily available clinical factors, age, baseline pure-tone audiometry, and cumulative drug dosage to predict hearing loss, covering a broad dosage range (20–90 g) and a significant post-treatment duration, which accounts for delayed drug elimination. It is particularly effective in detecting early-onset ultra-high-frequency hearing loss, a key marker of initial ototoxicity. This model was developed using a general patient population, enhancing its applicability for broader clinical use. Its simplicity allows potential integration into mobile apps, enabling quick predictions without additional tests, especially valuable in resource-limited settings (Adeyemo et al. 2024). For more information, refer to Ototoxicity Monitoring Guidelines (https://www.audiology.org/guideline-topic/ototoxicity-monitoring/).

### Developmental toxicity

It involves harmful effects of chemicals or exposure agents during prenatal or early postnatal periods on a developing organism, potentially resulting in growth retardation, congenital malformations, functional deficits, or fatal death. The Computer Assisted Evaluation of Industrial Chemical Substances for Registration, Evaluation, Authorisation and Restriction of Chemicals (CAESAR) QSAR models were developed to minimise false negatives, improving their suitability for Registration, Evaluation, Authorisation and Restriction of Chemicals (REACH) requirements. The CAESAR online platform ensures easy access for both industry and regulatory users to the developmental toxicity model and other endpoint models (Cassano et al. 2010). For more information, refer to OECD Test No. 421: Reproduction/Developmental Toxicity.

### Myotoxicity

It refers to harmful effects on muscle tissue, particularly skeletal muscles, which can result in weakness, soreness, inflammation (myositis), and in severe cases, extensive muscle breakdown. Using LASSO regression, nine key factors, including the skeletal muscle index, were identified and combined into a predictive model that was developed by (Yang et al. 2024). This model outperformed the skeletal muscle index alone, achieving strong predictive accuracy across training and validation groups. The results suggest that integrating radiomic features with conventional measures can improve risk prediction and aid in personalised treatment decisions (Yang et al. 2024).

### Osteotoxicity

It describes damage to bones caused by certain drugs, chemicals, or other harmful substances, which can result in bone thinning, structural weakness, slower healing, or even fractures in severe cases. BONEcheck is the first tool to use polygenic risk scores (PRS) for predicting fracture risk. Several PRSs have been developed based on genetic variants linked to bone mineral density or fractures, which incorporate 33 such variants. Although these scores were built from different sets of genes, they have all been validated as independent predictors of fracture risk beyond traditional clinical factors (Nguyen et al. 2023). According to the OECD, Test Guideline 414 provides guidance on assessing osteotoxicity.

### Enterotoxicity

It is often caused by drugs, chemicals, bacterial toxins, or other harmful agents that exert toxic effects on the intestines. It can damage the intestinal lining, disrupt normal absorption, and trigger inflammation (Duan et al. 2024). A web-based tool, ToxiM, predicts molecular toxicity, aqueous solubility, and permeability to build machine learning-based classification and regression models. These models achieved high accuracy (93%, MCC = 0.84) in 10-fold cross-validation and comparable performance on blind datasets. Validation with external datasets confirmed the tool’s reliability, making ToxiM a valuable resource for rapid toxicity and pharmacokinetic property prediction of small molecules (Sharma et al. 2017). Similarly, an enzyme-based tool, MicrobeRX designed to predict drug metabolites, incorporates 5487 human reactions, 4030 unique microbial reactions from 6286 genome-scale models, and 3650 drug metabolism reactions from DrugBank. It also provides modules for visualising metabolites and performing enzymatic and taxonomic analyses. Moreover, it outperformed BioTransformer 3.0 by offering better predictive accuracy, greater molecular diversity, and fewer redundant predictions (Ruiz-Moreno et al. 2025) . GutBug is a web-based tool that predicts bacterial metabolic enzymes capable of biotransforming xenobiotics and other molecules using a combination of machine learning, neural networks, and chemoinformatics. Trained on 3457 enzyme substrates and a curated database of 363,872 enzymes from about 700 gut bacterial strains, GutBug can accurately predict 0.78–0.97 across reaction types (Malwe et al. 2023). The tool is available at https://metabiosys.iiserb.ac.in/gutbug.

### Reproductive toxicity

Reproductive toxicity involves adverse effects on reproductive function or offspring development. It also refers to adverse effects of substances on any part of the reproductive cycle, including impaired reproductive functions and harmful impacts on embryos, such as growth retardation, malformations, functional impairments, or death (Toragall et al. 2022). To support reproductive toxicity prediction, Xpaths, a set of algorithms, were developed to enhance the reliability of compound effect assessments by linking test outcomes across species. It offers a user-friendly interface and provides open access to extraction, transformation, and loading scripts, along with enrichment algorithms that can be accessed from GitHub. These algorithms are applied to developmental and reproductive toxicity (DART) prediction within the DARTpaths platform (Bhalla et al. 2023). Under Regulation (EC) No. 1272/2008, the substance is considered to pose serious health risks, as it meets the criteria for classification as a carcinogen, a germ cell mutagen, or a reproductive toxicant (Categories 1 A, 1B, or 2).

### Endocrine toxicity

People are exposed to endocrine-disrupting chemicals (EDCs), which can lead to endocrine toxicity, even before birth. They are exposed through everyday products that contain synthetic chemicals. Many of these chemicals are not fully tested and may harm human health and the environment (Encarnação et al. 2019). EDCs can interfere with the endocrine system at multiple levels, affecting hormone synthesis, storage, and release, transport, metabolism, receptor binding, and post-receptor signaling. EDTox, a R Shiny application (Sakhteman et al. 2022), provides an intuitive graphical interface enabling non-expert R users to leverage large-scale toxicogenomics data for identifying and prioritising endocrine-disrupting compounds. It also facilitates exploration of molecular pathways selected by machine learning-based models. The tool is accessible as a standalone version via GitHub or as a web server https://doi.org/10.5281/zenodo.581709. For more information on endocrine toxicity, refer to clinical practice guidelines (https://www.endocrine.org/clinical-practice-guidelines).

### Cellular toxicity

CLC-Pred 2.0 is a web-based application for predicting cytotoxicity across human cell lines and associated molecular mechanisms. It accepts inputs such as SMILES, drug names, or drawn structures, providing probabilities of activity/inactivity (Pa/Pi) along with detailed descriptions. Results are downloadable, and prediction accuracy is validated through cross-validation. For instance, it correctly predicts erlotinib’s cytotoxicity against lung cancer cell lines, reflecting its known therapeutic use (Lagunin et al. 2023). Similarly, Cyto-Safe is a freely available web application http://insightai.labmol.com.br/ designed to identify potential cytotoxic compounds using QSAR-based machine learning models. It is built on a dataset of approximately 90,000 compounds tested against 3T3 and HEK 293 cell lines. Users can input SMILES strings, upload CSV or SDF files, or sketch molecular structures. The tool provides binary predictions for each cell line, confidence scores, and explainable AI (XAI) analyses (Feitosa et al. 2024). For more information, refer to OECD Test No. 491: short time exposure in vitro test method for identifying (i) chemicals inducing serious eye damage and (ii) chemicals not requiring classification for eye irritation or serious eye damage.

### Mitochondrial toxicity

Mitochondrial damage is caused by diseases or external substances, including environmental chemicals. It is not only associated with various illnesses but also plays a major role in drug-related toxic effects. The MitoTox database provides an approach for preclinical screening of mitochondrial toxicity, directly contributing to drug safety and public health. By examining different subtypes of mitochondrial toxicity, it seeks to clarify how changes in mitochondrial structure and function can signal the onset and progression of diseases. MitoTox is expected to enhance our understanding of mitochondria-related toxicity, open doors for early diagnosis of mitochondrial disorders, and support the development of innovative therapies (Lin et al. 2021). For more information, refer to OECD series on adverse outcome pathways No. 7 (Bal-Price et al. 2018).

###  Genotoxicity

*** Mutagenicity:*** The Ames mutagenicity test is the most widely used assay for estimating the mutagenic potential of drug candidates (Martínez et al. 2022). AMPred-CNN is a convolutional neural network model for predicting Ames mutagenicity. It significantly outperforms traditional ML models (e.g. RF, XGBoost) and recent ML/DL approaches, achieving an AUC of 0.954. The tool (https://nsclbio.jbnu.ac.kr/tools/AMPred-CNN/) leverages image-based molecular representations and is available as a user-friendly web service for toxicity and ADMET research (Tran et al. 2024).

For more information, refer, ICH GUIDELINE (https://www.ich.org/page/ich-guidelines)

*** Carcinogenicity:*** It includes both mutagenicity and carcinogenicity, which damage DNA and may lead to cancer**.** A deep learning-based model, DeepCarc (https://github.com/TingLi2016/DeepCarc), was developed to predict the carcinogenicity of small molecules. It was trained on 692 compounds and tested on 171 compounds from the NCTR liver cancer database, achieving a correlation of 0.432, outperforming four advanced DL-QSAR models by an average of 37%. It has also been applied to screen compounds from DrugBank and Tox21, offering a promising early detection tool for carcinogenicity assessment (Li et al. 2021). Similarly, CarcinoPred-EL is a web-based tool for predicting chemical carcinogenicity using three ensemble models (SVM, RF, XGBoost) trained on 1,003 compounds with rat carcinogenicity data. Among them, Ensemble XGBoost performed best, achieving ~70% accuracy, 67% sensitivity, and 76.5% specificity in external validation. The tool also screened potential carcinogens in the DrugBank database (Zhang et al. 2017) and is freely accessible at (http://112.126.70.33/toxicity/CarcinoPred-EL/about.html). For more information on carcinogenicity, refer to the regulatory guidelines of REACH, the substance qualifies for classification as a carcinogen (Category 1 A or 1B), germ cell mutagen (Category 1 A or 1B), or reproductive toxicant (Category 1 A, 1B, or 2) under Regulation (EC) No. 1272/2008.

*** Immunotoxicity:*** It refers to damage to the immune system caused by certain drugs, chemicals, or environmental toxicants. Such effects may either suppress immune defences, making the body more vulnerable to infections, or overstimulate the immune system, leading to allergies or autoimmune conditions (Zerdan et al. 2021). A computational model of immunotoxicity that is beyond traditional single-endpoint methods. By analysing data from an AhR activation assay in PubChem, which has built hundreds of QSAR models using a combinatorial approach and refined them into consensus models by averaging predictions. From these, 20 top-performing assays were chosen, which helped identify important immune-related effects such as Pregnane X Receptor (PXR), Retinoic Acid Receptor (RAR) activation, Androgen Receptor (AR), Progesterone Receptor (PR), and Thyroid Receptor (TR) inhibition, mutagenicity, and mitochondrial damage. These models effectively predicted various immunotoxicants, including pesticides and pollutants. This approach provides a valuable tool for evaluating complex toxic effects, even when available data is limited (Daood et al. 2024). For more information, refer to OECD, test guideline No. 444 A on in vitro immunotoxicity.

*** Enzyme Inhibition:*** Chemical compounds that suppress the activity of key metabolic enzymes, such as CYP1A2, CYP2C19, CYP2C9, CYP2D6, CYP3A4, and CYP2E1. CypReact is a computational tool designed to predict whether a molecule will interact with one of nine major human CYP450 enzymes using SMILES or SDF inputs. It achieved AUROC scores of 0.83–0.92, outperforming tools like ADMET Predictor and SMARTCyp, making it a valuable resource for in silico metabolism prediction in toxicity assessment (Tian et al. 2018). It is freely available at https://bitbucket.org/Leon_Ti/cypreact/src/master/. BioTransformer (https://biotransformer.ca) is a freely accessible web server for rapid and comprehensive in silico metabolism prediction. Compared to earlier versions, BioTransformer offers 40–50% higher accuracy, improved multi-step transformation simulations, and broader metabolite coverage, making it a powerful tool for metabolism studies and toxicity assessment (Wishart et al. 2022). For more information, refer to OECD test guideline No. 456 H295R steroidogenesis assay and OECD test guideline 457—National Toxicology Program.

## Molecular descriptors (MD) for toxicity prediction

Molecular descriptors play a crucial role in predicting chemical toxicity based on the structural, physicochemical, and electronic properties of chemicals (Table 3). These descriptors translate complex molecular structures into quantifiable features that can be analysed by computational models. They serve as the foundation for Quantitative Structure–Toxicity Relationship (QSTR) models, which correlate specific molecular features with known toxicological outcomes (Carracedo-Reboredo et al. 2021). Molecular descriptors are essential tools in modern computational toxicology, enabling more efficient, ethical, and reliable assessments of toxicity. By quantifying structural features such as hydrophobicity, molecular size, and electronic reactivity, these descriptors help in identifying the potential toxic functional groups, aka ‘toxicophores’ that are present in the molecule. For instance, the neurotoxic chemicals such as aldicarb, diazinon, and phosmat can be analysed based on their molecular descriptors. In a study by Jiang et al. (2020), descriptors that were used to correlate with neurotoxicity are Morgan autocorrelation descriptors (MATSe3 and MATSv3), E-state descriptors (Smin32 and Smin8), and hydrophilicity index (Hy). Among them, MATSe3, representing atomic Sanderson electronegativity weighting, has the highest contribution towards neurotoxicity, while ‘Hy’ is inversely correlated with neurotoxicity. Similarly, for cardiotoxicity assessment, approximately 20,000 compounds were analysed, which showed both toxic and non-toxic molecules, sharing descriptors such as molecular weight, volume, number of hydrogen donors and acceptors, total polar surface area (TPSA), and LogP. It can be interpreted that compounds that have TPSA in the range of 50 to 100, and hydrogen bond acceptors between 6 and 10, may be indicative of toxicity (Liu et al. 2025). A few commonly used molecular descriptor calculation tools are shown in Table 3.

**Table 3:** Molecular descriptor prediction tools

Sl No.	Tools	Description	URL	References
1	VEGAHUB	A free platform providing numerous QSAR models for various environmental and ecotoxicological endpoints. In recent years, it has expanded to include additional tools for read-across and prioritisation, which can be applied in an integrated manner	https://www.vegahub.eu/	(Roncaglioni et al. 2022)
2	ChemDes	An easy-to-use platform for computing molecular descriptors and fingerprints, enabling efficient data analysis, visualisation, and predictive modeling across applications	http://www.scbdd.com/chemdes/	(Dong et al. 2015)
3	PaDEL	PaDEL-Descriptor is software for calculating 797 molecular descriptors (663 1D/2D and 134 3D) and 10 types of fingerprints, using the Chemistry Development Kit, with additional features like electrotopological states, McGowan volume, and substructure-based descriptors	https://github.com/ecrl/padelpy	(Yap 2011)
4	MOLE DB	A free web-based database containing 1,124 molecular descriptors calculated for 234,773 molecules from the NCI database. It serves as a research and teaching tool, enabling users to search molecules, analyse descriptor values, and export data	https://www.moldb.com/	(Ballabio et al. 2009)
5	Mordred	A user-friendly molecular descriptor calculation tool that generates over 1,800 descriptors with high computational efficiency. It can be installed with a single command, offers a web interface, and is distributed under a 3-clause BSD license	https://github.com/mordred-descriptor/mordred	(Moriwaki et al. 2018)
6	QSARINS	A software for analysis of structure–activity relationships for environmental and ecotoxic chemicals	https://dunant.dista.uninsubria.it/qsar	(Gramatica et al. 2013)
7	KNIME	Konstanz Information Miner is a open-source visual programming platform that does not need programming expertise	https://www.knime.com/	(Berthold et al. 2007)
8	RDKit	Open-source toolkit for cheminformatics and machine learning used for molecular descriptor calculation, QSAR modelling and property prediction	https://www.rdkit.org/	-

## Quantitative Structure–Toxicity Relationship (QSTR)

Structure–Activity Relationship (SAR) investigates the compound’s chemical structure and its biological effects based on the principle that a molecule’s structural features play a crucial role in determining its biological activity/toxicity (Guha 2013)**.** Quantitative Structure–Toxicity Relationship (QSTR) approaches have been increasingly utilised to reduce the number and cost of animal experiments (Singh et al. 2014). For instance, QSTR models provide valuable insights into hepatic biotransformation by revealing how structural features of xenobiotics relate to their metabolic pathways. Such analyses enhance the understanding of biochemical mechanisms and enzymatic activities, enabling the identification of critical metabolic hotspots and potential toxicity sites (Rai et al. 2023). Similarly, QSTR models were also constructed for *Daphnia magna*, fish, and algal species using partial least squares (PLS) regression with multiple variables to predict the toxicity of cationic polymers (Khan et al. 2021). Some of the commonly used QSTR tools are described in Table 4.

**Table 4:** Quantitative Structure–Toxicity Relationship (QSTR) tools

Sl No.	Tools	Description	References
1	OECD QSAR Toolbox	A free tool designed to help group chemicals based on their structural and mechanistic similarities. It also provides detailed reports for each prediction, covering the underlying mechanisms, reference chemicals, statistical analysis, applicability, and validity	(Yordanova et al. 2019)
2	QSAR-Co	A free and user-friendly standalone software designed to develop robust classification-based QSAR models using two widely applied techniques, Linear Discriminant Analysis (LDA) and Random Forest (RF)	(Ambure et al. 2019)
3	QSAR‑Co‑X	A user-friendly, open-source toolkit built in Python, designed to enhance and expand the capabilities of the earlier Java-based QSAR-Co for multitasking QSAR (mt-QSAR) modeling	(Halder and Dias Soeiro Cordeiro 2021)
4	DTC-QSAR	An all-in-one modeling software with a simple, user-friendly interface for building both regression (MLR, PLS) and classification (LDA, Random Forest) QSAR models	(Roy 2019)
5	Online Chemical Modeling Environment (OCHEM)	A free, web-based platform designed to automate QSAR modeling, supporting QSAR/QSPR model development from data search and descriptor calculation to machine learning model building, validation analysis	(Sushko et al. 2011)
6	chemprop	A versatile machine learning toolbox for predicting the chemical properties of molecules and reactions, offering extensive customisation options such as user-defined atom and bond features, and multiple loss functions	(Heid et al. 2024)
7	Coral	An interactive analysis tool for creating and visualising cohorts based on various attributes, allowing users to explore cohort evolution, assess prevalences, and access detailed single-item information	(Adelberger et al. 2021)
8	Rm square	Directly measures the difference between observed and predicted values, offering a more stringent assessment of model predictivity. The rm^2^ metric has three forms: rm^2^ (LOO) for internal validation, rm^2^ (test) for external validation, and rm^2^ (overall) for combined evaluation	(Roy and Mitra 2012)
9	VCCLAB	a multi-platform, web-based software system designed to enable computational chemists to perform a wide range of molecular property calculations and data analyses	(Kobayashi et al. 2021)
10	Ecosar	The widely used ECOSAR tool for QSAR-based prediction of aquatic toxicity was evaluated using experimental acute toxicity data from over 1,000 industrial chemicals tested on fish, Daphnia, and green algae. When a tolerance factor of 10 was applied, the prediction accuracy improved to 69% for fish, 64% for Daphnia, and 60% for algae	(Reuschenbach et al. 2008)

## Artificial intelligence/machine learning (AI/ML) models in toxicity prediction

In recent years, AI/ML have gained significant attention for their ability to analyse large datasets and identify complex patterns related to chemical toxicity. AI/ML models are increasingly used to predict chemical toxicity. Machine learning tools and models such as support vector machines, random forests, and deep learning are being used to process complex datasets and enhance prediction accuracy (Tables 5 and 6). These models target various endpoints such as acute oral toxicity, hepatotoxicity, cardiotoxicity, and mutagenicity (Fig. 3). However, their performance varies across datasets due to differences in complexity, class balance, and chemical space, making comparisons difficult (Cavasotto and Scardino 2022). To ensure trust within regulatory environments, it is crucial that AI-based tools are interpretable and their decision-making processes are transparent. Regulatory agencies are increasingly adapting their frameworks to support the safe and effective integration of AI/ML in the drug development process. For instance, the U.S. FDA is developing a flexible, risk-based regulatory structure to promote innovation. Similarly, the European Union’s proposed Artificial Intelligence Act aims to set unified, risk-based regulations for AI development and use, with emphasis on ethical implementation; though challenges such as limited expertise and potential inefficiencies in oversight remain (Niazi 2025; Pasas-Farmer and Jain 2025).

**Table 5:** Machine learning models in toxicity prediction

Sl. No.	Algorithm/model	Endpoint	Reference
1	Linear Regression	Endocrine toxicity	(Sharma et al. 2017; Sakhteman et al. 2022)
2	Polynomial regression	All toxicity endpoints	(Mansouri et al. 2021)
3	Logistic Regression	Animal toxicity	(Gomes et al. 2025; Ryu et al. 2023)
4	Decision tree	All toxicity endpoints, neurotoxicity	(De Sá et al. 2022; Sharma et al. 2017; Iftkhar et al. 2022)
5	Lasso	All toxicity endpoints	(Mansouri et al. 2021)
6	Random Forest	Animal toxicity, neurotoxicity, Pulmonary toxicity, carcinogenicity, Cytochrome inhibition, ecotoxicity	(De Sá et al. 2022; Sharma et al. 2017; Iftkhar et al. 2022; Mayr et al. 2016; Borba et al. 2022; Tieghi et al. 2025; Shin et al. 2022; Gomes et al. 2025; Arab and Barakat 2021; Ryu et al. 2023; Pang et al. 2025; Shehab et al. 2025; Zhang et al. 2017; Plonka et al. 2021; Banerjee et al. 2020)
7	SVM	Neurotoxicity, hepatotoxicity, carcinogenicity	(De Sá et al. 2022; Sharma et al. 2017; Mayr et al. 2016; Tieghi et al. 2025; Shin et al. 2022; Gomes et al. 2025; Arab and Barakat 2021; Pang et al. 2025; Zhang et al. 2017)
8	KNN	All toxicity endpoints	(De Sá et al. 2022; Sharma et al. 2017; Gomes et al. 2025)
9	Gradient Boosting	All toxicity endpoints, cardio toxicity	(De Sá et al. 2022; Iftkhar et al. 2022; Tieghi et al. 2025)
10	Hierarchical Clustering	Human toxicity	(Hasan et al. 2025)
11	Density-Based Spatial Clustering of Applications with Noise (DBSCAN)	Cytotoxicty	(Sayed et al. 2024)
12	Principal Component Analysis	Entereotoxicity	(Sharma et al. 2017)
13	Linear Discriminant Analysis	Genotoxicity	(Gomes et al. 2025)
14	Uniform Manifold Approximation and Projection (UMAP)	Peptide toxicity	(Guan et al. 2025)
15	Semi-Supervised Learning	All toxicity endpoints	(Chen et al. 2021)
16	Generative Adversarial Networks (GAN)	Genotoxicity	(Chen et al. 2022)
17	Reinforcement Learning	All toxicity endpoints	(Sharma et al. 2023)
18	Ensemble Learning	Aquatic toxicity, Fish toxicity, cellular toxicity, carcinogenicity	(Shi et al. 2024; Peets et al. 2022; Feitosa et al. 2024; Zhang et al. 2017)
19	Neural Networks	Neurotoxicity, cardiac toxicity, hepatotoxicity	(Banerjee et al. 2024, 2020; De Sá et al. 2022; Han et al. 2025; Tran et al. 2024; Gomes et al. 2025; Chen et al. 2021; Pang et al. 2025; Karim et al. 2021)
20	Deep Learning	Carcinogenicity, immunogenicity	(Arab and Barakat 2021; Li et al. 2021; Mayr et al. 2016; Mustafa et al. 2025)
21	Elastic Net Regression	All toxicity endpoints	(Mayr et al. 2016)
22	Naïve Bayes	Dermal toxicity, cellular toxicity	(Tieghi et al. 2025; Gomes et al. 2025; Borba et al. 2021; Lagunin et al. 2023)
23	Hidden Markov Models (HMM)	Genotoxicty	(Díaz-Valerio et al. 2021)
24	Monte Carlo	Nephrotoxicity	(Toropov et al. 2023)
25	Genetic Algorithms	Ecotoxicity	(Gao et al., 2022)
26	Non-Negative Matrix Factorization (NMF)	Ecotoxicity	(Mei 2024)
27	Hybrid Methods	Animal toxicity	(Limbu et al. 2022; Pang et al. 2025)
28	Long short-term memory (LSTM)	Peptide toxicity	(Gao et al. 2025)
29	Genetic programming	Aquatic toxicity	(Searson et al. 2011)
30	Recurrent Neural Networks	Molecular toxicity	(Peng et al. 2020)
31	Transformers	Multi-organ toxicity	(Ha et al. 2025)
32	t- Distributed Stochastic Neighbour Embedding (t-SNE)	All toxicity endpoints	(Sosnin 2024)
33	Gaussian process	Metabolism toxicity	(Setiya et al. 2024)
34	Bidirectional Encoder Representations from Transformers (BERT)	Environmental toxicity	(Tan et al. 2024)
35	Linear discriminant analysis	Fish toxicity	(Martin et al. 2013)
36	Generative pre-trained transformer	All toxicity endpoints	(Xu et al. 2025)
37	Language model	Hepatotoxicity, cardiotoxicity, mutagenicity	(Bran et al. 2024)
38	Autoencoder	Protein toxicity	(Shi et al. 2022)
39	Gated Recurrent Units-DNN	Cardiotoxicity	(Ylipää et al. 2023)
40	Bagging	Mitochondiral toxicity	(Igarashi et al. 2024)

**Table 6:** Tools for toxicity prediction

S. No.	Tool name	Application	Link	References
1	EDTox	Endocrine disruptors prediction	https://doi.org/10.5281/zenodo.5817093)	(Sakhteman et al. 2022)
2	DTreePred	Assessing the pathogenicity of nucleotide variants	https://danhfg.github.io/dtreepred/	(Gomes et al. 2025)
3	PredAOT	Acute oral toxicity prediction	https://predaot.netlify.app/	(Ryu et al. 2023)
4	ToxCSM	Small molecule toxicity prediction	http://biosig.lab.uq.edu.au/toxcsm	(De Sá et al. 2022)
5	CardioToxCSM	Cardiotoxicity prediction	https://biosig.lab.uq.edu.au/cardiotoxcsm/	(Iftkhar et al. 2022)
6	Protox 3.0	Small molecule toxicty precition	https://tox.charite.de/protox3/	(Banerjee et al. 2024)
7	DeepTox	Toxicty prediction	https://www.bioinf.jku.at/	(Mayr et al. 2016)
8	StopTox	Acute toxicity prediction	https://stoptox.mml.unc.edu/	(Borba et al. 2022)
9	ToxSTAR	Drug-induced liver injury prediction	https://www.kitox.re.kr/toxstar/	(Shin et al. 2022)
10	ToxTree	Toxicity prediction	https://toxtree.sourceforge.net/	(Arab and Barakat 2021)
11	NeuTox	Neurotoxicity prediction	https://github.com/xuejunhe/NeuTox-2.0	(Pang et al. 2025)
12	CarcinoPred-EL	Carcinogenicity prediction	http://112.126.70.33/toxicity/CarcinoPred-EL/about.html	(Zhang et al. 2017)
13	SuperCYPsPred	Cytochrome activity prediction	https://www.scinapse.io/papers/3011211676	(Banerjee et al. 2020)
14	ToxiPep	Peptide toxicity prediction	https://awi.cuhk.edu.cn/~dbAMP/ToxiPep/	(Guan et al. 2025)
15	AquaticTox	Aquatic toxicity prediction	https://chemyang.ccnu.edu.cn/ccb/server/AquaticTox	(Shi et al. 2024)
16	MS2Tox	Ecotoxicity prediction	https://github.com/kruvelab/MS2Tox	(Peets et al. 2022)
27	Cyto-Safe	Cytotoxicity prediction	http://insightai.labmol.com.br/	(Feitosa et al. 2024)
18	HepatoToxicity Portal	Hepatoxicty prediction	https://kobic.re.kr/htp/	(Jin et al. 2023)
19	DeepCarc	Carcinogenicity prediction	https://github.com/TingLi2016/DeepCarc	(Li et al. 2021)
20	PredSkin	Dermatopxicty prediction	https://bio.tools/pred-skin	(Borba et al. 2021)

## Explainable AI (XAI)

The emergence of AI is significantly accelerating and refining toxicity prediction methods for environmental safety and protection (Qadri et al. 2025). DL models have been widely used in ecotoxicity prediction, demonstrating significant promise in the field. However, they also pose a significant challenge, widely known as the ‘black-box’ problem, due to the opacity of the model's inner workings. The mathematical concepts underlying the models can often be difficult for humans to interpret. The challenge arises because the researchers and regulators need to understand how the model arrived at a conclusion. This black-box prediction makes it difficult to validate the recommendations (Ramakrishnan et al. 2025). In simple terms, a gap exists between the prediction logic of AI and the need to understand the rationale behind the prediction. This necessitates a demand for ‘explainable’ deep learning methods to address the narrative and to comply with regulatory requirements (Jiménez-Luna et al. 2020). The AI models are trained on datasets that inherently contain bias. This may result in misleading predictions, whereas XAI provides transparency and confidence in the model for researchers and regulators by detecting and correcting the bias, thereby ensuring the reliability of the model prediction (Qadri et al. 2025). AI can accelerate the toxicity prediction, and XAI ensures that these predictions are interpretable, and reduces the false positives at an early stage. This helps streamline the process, leading to faster and more reliable advancements.

## Challenges and limitations

Toxicity prediction is often resource-intensive, time-consuming, and associated with ethical considerations (Yang et al. 2018). To overcome these challenges, computational methods are being adapted to allow researchers to assess toxicological risks. These methods include a variety of techniques such as quantitative structure–activity relationships (QSAR), read-across strategies, and pharmacokinetic modelling (Ahlberg et al. 2014). In recent years, AI/ML approaches have facilitated various toxicity predictions (Cavasotto and Scardino 2022). Although computational and AI/ML models have made significant strides in toxicity prediction, several limitations remain. A major concern is the quality and availability of toxicological data, which can be incomplete, inconsistent, or poorly documented. This not only impacts model training but also reduces reliability. Additionally, many datasets are imbalanced, with a large proportion of non-toxic samples, which can potentially lead to biased predictions. A major challenge is the lack of high-quality, consistent and sufficiently large datasets, making it difficult to develop reliable toxicity prediction models (Guo et al. 2023). Another challenge is that these models often do not perform well outside the specific chemical datasets on which they were trained, making it difficult to apply them to new chemicals. The lack of standardised methods for building and validating models also makes it difficult to compare and reduces confidence in the predictive performance. Furthermore, many models are purely data-driven and do not consider the underlying biological mechanisms, which can lead to inaccurate predictions when dealing with complex biological systems. Due to these issues, regulatory agencies remain cautious about using AI-based models, primarily because of concerns over transparency, reproducibility, and the lack of robust validation frameworks. To improve accuracy, better integration of diverse data sources is needed through standardised preprocessing and multimodal methods like ensemble methods or federated learning. Additionally, the limited interpretability of black-box models remains a concern, highlighting the importance of explainable AI tools to improve confidence (Hassija et al. 2024). The EU REACH Regulation (EC No 1907/2006) is a leading global framework for chemical safety. It requires registration of chemicals produced or imported in quantities over 1 tonne per year, along with risk assessment, authorisation of Substances of Very High Concern (SVHCs), and specific restrictions managed by the European Chemicals Agency (ECHA) (Andreassen et al. 2025). Most countries regulate AI under the Software as a Medical Device (SaMD) framework. The regulations vary between binding legislation (hard law) and voluntary guidelines/codes of conduct (soft law). Several countries have made significant strides in developing policies and statutory frameworks to regulate the use of AI (Palaniappan et al. 2024). In the USA, the Food and Drug Administration (FDA) utilises a SaMD framework, emphasising Good Machine Learning Practices (GMLP), transparency, and bias elimination. In the UK, National Institute for Health and Care Excellence (NICE) and the Medicine and Healthcare products Regulatory Authority (MHRA) frameworks focus on cybersecurity and evolving algorithms (Mehta et al. 2025). In the European Union, moving from soft-law ethics guidelines to a binding AI Act, a risk-based approach classifies healthcare AI as ‘high-risk’, whereas in Australia, the Therapeutic Goods Administration (TGA) regulates SaMDs with a risk-based classification; the TGA provides guidelines on high-risk classification, while low-risk classification is largely exempted. In China, National Medical Products Administration (NMPA) guidelines for AI-aided software apply risk-based oversight. In Brazil, the drafted AI law classifies healthcare AI as ‘high-risk’, which requires public risk assessments and provider liability. In Singapore, the National AI Strategy and AI Verify governance toolkit; Health Sciences Authority (HSA) lifecycle guidelines and Ministry of Health (MOH) healthcare-specific AI guidelines (Palaniappan et al. 2024). Limitations on regulations differ widely across countries and jurisdictions, with a notable lack of convergence. Current frameworks are inclined towards providing guidelines on SaMD; however, the continuously evolving capabilities of AI, which include autonomous learning and adaptation, challenge existing rules. Ongoing discussions on regulatory guidelines are currently focused on achieving global alignment. Regulatory adaptability is key as AI evolves rapidly.

## Conclusion and future perspectives

AI/ML models are becoming increasingly vital for assessing the safety of chemicals and their effects on human health and the environment (Kim and Choi 2025). Future research is expected to focus on integrating AI/ML models with a systems-level approach to improve the mechanistic understanding of toxicity and enhance the biological relevance of predictive models (Guo et al. 2023). Another promising frontier is the rise of explainable artificial intelligence (XAI), which addresses one of the main limitations of deep learning models, their ‘black-box’ nature. Although these models often achieve high predictive accuracy, their lack of interpretability hinders regulatory acceptance (Hartung 2023; Jia et al. 2023). Overall, the integration of computational tools and AI/ML models is transforming the landscape of toxicology. These technologies offer faster, cost-effective, and ethically sound alternatives to traditional animal-based testing by leveraging large-scale datasets and advanced algorithms to generate reliable toxicity predictions. Conventional techniques such as QSAR modelling, machine learning, deep learning, and read-across have shown strong potential in identifying toxic compounds and their mechanisms of action. Advances in explainable AI, multi-omics integration, and mechanism-based modelling help address persistent challenges related to data quality, model transparency, and regulatory acceptance. Despite ongoing hurdles in standardisation and external validation, continued collaboration among computational scientists, toxicologists, and regulatory authorities will be essential to achieving meaningful progress. For researchers, the ethical and efficient integration of AI can significantly accelerate various stages of toxicity prediction. For the AI to learn, it requires a vast amount of data, which can be either already available or generated. Since the learning is dependent on the data and scenarios, the role that the data plays is very significant. Researchers should ensure that the data used to train is unbiased. Historically, toxicity prediction has been based on studies conducted on animals. Using AI for this task presents a distinct set of challenges. Practitioners play a key role in collecting and generating data. Although practitioners may not require a large amount of data for their day-to-day operations, their insights and experience play a vital role in how the models learn. AI can help practitioners in various capacities. It can act as decision support and as a bookkeeping assistant. AI as a virtual assistant is becoming widely popular due to its accessibility and ability to learn continuously. Like researchers, policymakers also work with large amounts of data. The data that policymakers examine is different from that of researchers. The comments and feedback on the various policies play a vital role in designing new policies and amending existing ones. AI can contribute by identifying hidden trends or searching for patterns that are not typically identifiable through manual work. Ultimately, AI-driven computational toxicology is poised to redefine chemical safety evaluation by enabling reliable, human-relevant, and animal-free approaches that will shape the future of toxicological research and regulation.

## Data Availability

This manuscript has no associated data.
